# Surgical and oncological management of renal medullary carcinoma in a young patient: a case report

**DOI:** 10.3389/fonc.2023.1073728

**Published:** 2023-05-02

**Authors:** Jean Courcier, Alexandre De La Taille, Riccardo Bertolo, Daniele Amparore, Selcuk Erdem, Onder Kara, Michele Marchioni, Nicola Pavan, Eduard Roussel, Maria Mamodaly, Riccardo Campi, Alexandre Ingels

**Affiliations:** ^1^ Department of Urology, Henri Mondor Hospital, University of Paris Est Créteil (UPEC), Créteil, France; ^2^ Université Paris-Saclay, CEA, CNRS, Inserm, BioMaps, Villejuif, France; ^3^ Department of Urology, San Carlo Di Nancy Hospital, Rome, Italy; ^4^ Division of Urology, Department of Oncology, School of Medicine, San Luigi Hospital, University of Turin, Orbassano, Italy; ^5^ Division of Urologic Oncology, Department of Urology, Istanbul University Istanbul Faculty of Medicine, Istanbul, Türkiye; ^6^ Department of Urology, Kocaeli University School of Medicine, Kocaeli, Türkiye; ^7^ Urology Unit, Department of Medical, Oral and Biotechnological Sciences, "G. d'Annunzio" University of Chieti, Chieti, Italy; ^8^ Urology Clinic, Department of Medical Surgical and Health Science, University of Trieste, Trieste, Italy; ^9^ Department of Urology, Onze-Lieve-Vrouwziekenhuis (OLV) Hospital, Aalst, Belgium; ^10^ Department of Urology, University Hospitals Leuven, Leuven, Belgium; ^11^ Pathology Department, University of Paris Est Créteil (UPEC), Henri Mondor Hospital, Créteil, France; ^12^ Unit of Urological Robotic Surgery and Renal Transplantation, Careggi Hospital, University of Florence, Florence, Italy; ^13^ Department of Experimental and Clinical Medicine, University of Florence, Florence, Italy

**Keywords:** non-clear cell RCC, renal medullary carcinoma, SMARCB1, chemotherapy, onco-urological management

## Abstract

Renal medullary carcinoma (RMC) is a rare form of renal cell carcinoma that has a poor prognosis. It is known to be associated with sickle cell trait or disease, although the exact underlying mechanisms are still unclear. The diagnosis is made through immunochemical staining for SMARCB1 (INI1). In this report, we present a case of a 31-year-old male patient with sickle cell trait who was diagnosed with stage III right RMC. Despite the poor prognosis, the patient survived for a remarkable duration of 37 months. Radiological assessment and follow-up were primarily performed using 18F-FDG PET/MRI. The patient underwent upfront cisplatin-based cytotoxic chemotherapy before surgical removal of the right kidney and retroperitoneal lymph node dissection. Identical adjuvant chemotherapy was administered post-surgery. Disease relapses were detected in the retroperitoneal lymph nodes; these were managed with chemotherapy and surgical rechallenges. We also discuss the oncological and surgical management of RMC, which currently relies on perioperative cytotoxic chemotherapy strategies, as there are no known alternative therapies that have been shown to be superior to date.

## 1 Introduction

Renal carcinoma represents approximatively 2%–3% of solid cancers. Clear cell renal cell carcinoma is the most common type, accounting for 75% of cases, followed by papillary renal cell carcinoma (10%) and chromophobe renal cell carcinoma (<5%) (1). There are other rare pathological entities that present with a wide diversity of histological types. Among these rare entities is renal medullary carcinoma (RMC), which accounts for approximatively 0.5% of renal carcinoma (2). RMC mostly affects men (gender ratio: 2:1), and most commonly affects adolescents and young adults (median age at diagnosis: 28 years), with the right kidney being preferentially involved (3). This pathology is almost exclusively associated with heterozygous sickle cell trait or disease and other hemoglobinopathies (4). Thus, the practice of high-intensity exercise in such patients could hypothetically be a risk factor (5). The diagnosis is commonly made by investigating general symptoms such as abdominal pain, fatigue, weight loss, and, more specifically, by gross hematuria or clinical palpation of a flank or abdominal mass (6). The disease is more likely to be diagnosed at an advanced or metastatic stage (7). In addition, several reports have described rapid metastatic dissemination even in patients who were treated early for localized RMC (4, 6, 8). Radiological evaluation of the disease relies on enhanced computed tomography (CT), which typically shows a weakly and heterogeneously enhanced tumor, a central localization in the kidney with respect of the kidney’s outline. (9, 10). The radiological aspect on CT scan can mimic a urothelial upper tract tumor invading the renal parenchyma. This medical condition has a poor prognosis, with a reported median overall survival (OS) of 13 months (95% CI: 9.0–17.9 months) (6). The physiopathology of RMC is still hypothetical, but chronic stress hypoxia associated with sickle cell disease is suspected to play a role (11). The first histological characterization of this cancer dates back to 1995 (12). On histopathological examination, an infiltrative tumor is readily visible, originating from the medullary boundary with the kidney’s excretory tract. The tumor is composed of poorly differentiated cells and shows neutrophil infiltration. Cystic components may also be present. Finally, the loss of expression of SMARCB1 (INI1) is suspected to play a central role in the pathogenesis of RMC, and confirmation of this loss of expression through immunohistochemistry confirms the diagnosis (13–16). In this report, we present a case of the management of RMC.

## 2 Case presentation

**Figure 1:**
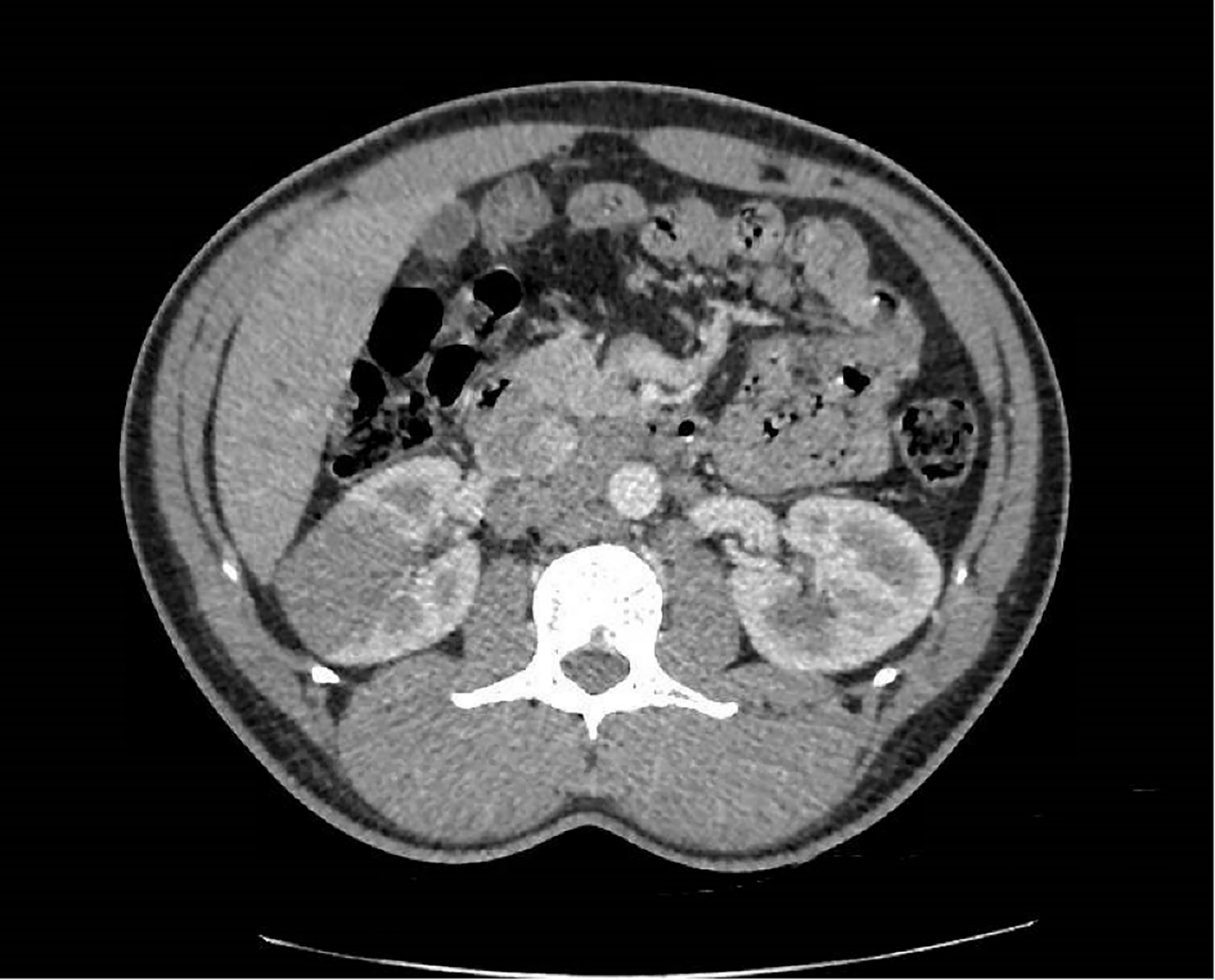
Axial enhanced CT scan showing the poorly delimited right renal mass at diagnosis.

In April 2019, a 31-year-old male patient was referred by a practitioner for evaluation of right lower back pain that had been ongoing for 3 months, along with weight loss of 6 kg over a 6-month period. The patient reported experiencing a sense of heaviness in the right hypochondrium towards the end of 2018, accompanied by colicky abdominal pain that increased in intensity over the following months. However, no symptoms of altered bowel movements or hematuria were reported. The patient had a history of heterozygous sickle cell trait and had suffered from malaria infection at the age of 12. He had no other significant medical history and reported no family history of neoplasia. Abdominal ultrasound imaging revealed a 50-mm mass in the patient’s right kidney. Subsequent CT scans showed a heterogeneous 50 × 47 × 45 mm parenchymal renal mass involving the renal sinus (**Figure 1**), along with lymph node involvement in para-aortic and inter-aortocaval locations. There was no extension to the renal vein, and no abnormalities were found in the liver or at the thoracic or skeletal levels.

Whole-body 18F-FDG PET/MRI revealed a 43-mm hypermetabolic lesion in the right kidney, which was accompanied by hypermetabolic retroperitoneal lymph node invasion and a single hypermetabolic lymph node above the diaphragm in the left sub-clavicular position. There was no evidence of invasion in the viscera or bones.

The diagnosis of RMC was established based on a biopsy of the renal mass: analysis of eight biopsy samples revealed proliferation of a poorly differentiated carcinoma tumor consisting of basophilic cellular elements with increased nuclear–cytoplasmic ratio, irregular hyperchromatic nuclei, and frequent mitotic figures. The proliferation exhibited a trabecular-cord architecture, and there was no evidence of glandular inflection. It was located within a highly inflammatory stroma, and the entire tumor was separated by sclerohyaline areas in which residual renal tubules and numerous congestive vessels without embolism were observed. There was no evidence of a lymphomatous process. Immunohistochemical analysis revealed positive staining of the tumor cells for Vimentin, EMA, CK7, PAX8, and E-Cadherin, and negative staining for CD117, CD10, CK20, CK5-6, P40, and GATA 3. These immunohistochemical findings excluded the possibility of urothelial carcinoma. The histological appearance was consistent with an epithelial tumor of renal origin. The pathological report further indicated tumor proliferation, with an immunohistochemical profile (SMARCB1/INI1 negative) consistent with the proposed diagnosis of RMC. Notwithstanding, 20% of tumoral cell membranes were positive for PD-L1 staining in immunohistochemistry.

At the time of diagnosis, the patient’s general condition was good, with a body mass index of 25.6 kg/m^2^ and a performance status of 0. The case was discussed in a multidisciplinary meeting, and systemic therapy was recommended. The initial treatment plan included three cycles of paclitaxel (80 mg/m^2^ on days 1 and 8), gemcitabine (1,000 mg/m^2^ on days 1 and 8), and cisplatin (70 mg/m^2^ on day 1), given in 21-day cycles. The patient commenced chemotherapy in June 2019.

After completion of three cycles of chemotherapy, 18F-FDG PET/MRI showed a partial response, with reduction in the size of the primary tumor from 43 mm to 26 mm, and no uptake observed in the supra-diaphragmatic lymph node. However, a hypermetabolic 23-mm inter-aortocaval lymph node and multiple weakly metabolic retroperitoneal nodes smaller than 1 cm were still present.

In September 2019, the patient underwent an open right nephrectomy with retroperitoneal lymph node dissection, which took 180 min and resulted in an estimated blood loss of 700 ml. No post-operative complications of grade higher than CLAVIEN-DINDO level 1 were reported.

**Figure 2:**
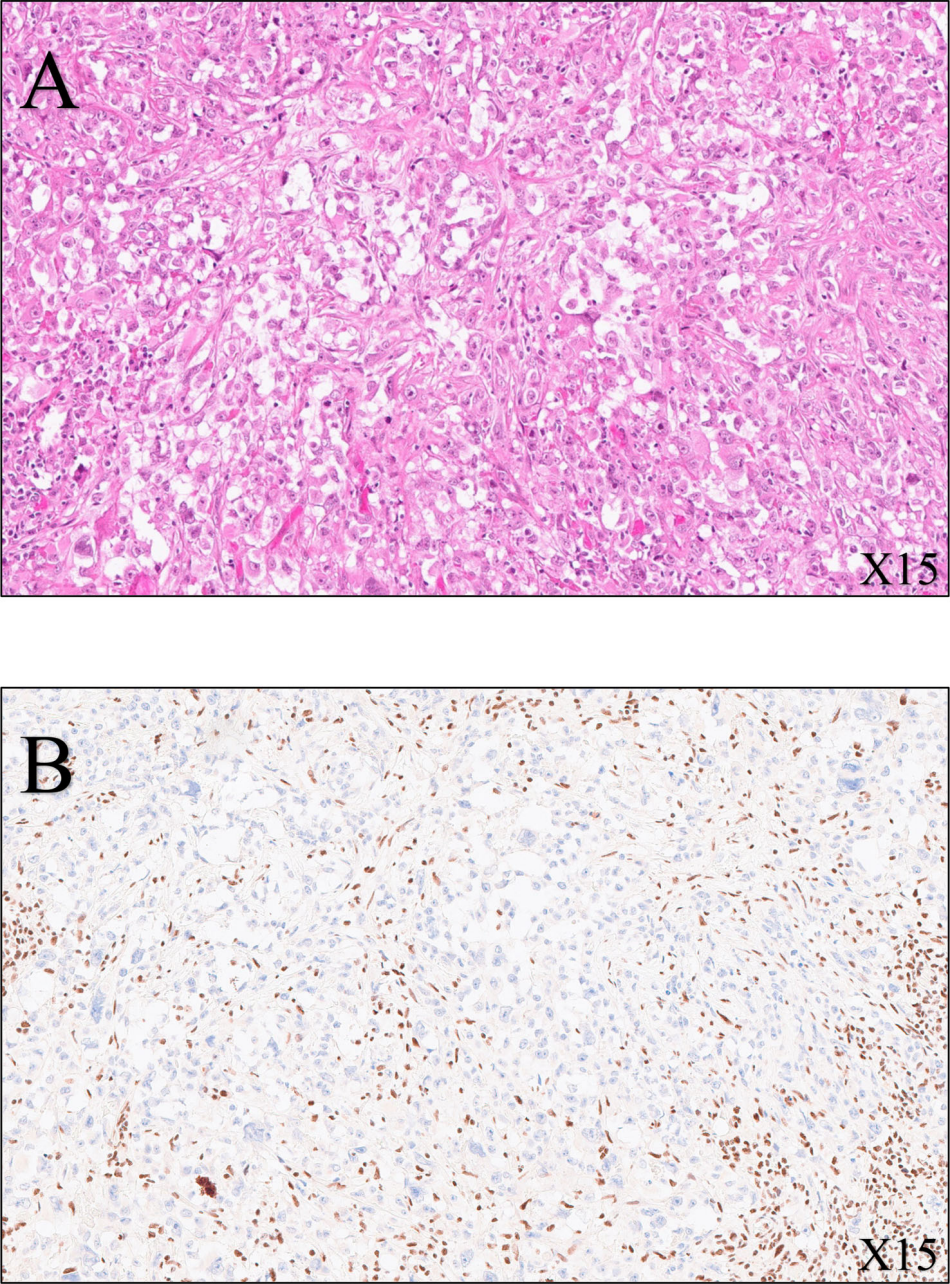
Histological features of the radical nephrectomy of the right kidney. **(A)** SMARCB1-deficient renal medullary carcinoma (hematoxylin–eosin–saffron, ×15 magnification): tumor cells are pleomorphic with enlarged nuclei, vesicular chromatin, proeminent nucleoli, and eosinophilic cytoplasm. **(B)** Immunohistochemistry (SMARCB1, ×15 magnification) shows loss of expression of SMARB1 (also known as INI1, SNF5, or BAF47) within the tumor cells, whereas intratumoral lymphocytes are strongly positive (nuclear stain).

Histological examination revealed a remnant of RMC with evidence of a therapeutic response, as indicated by 60% fibrotic involution of the renal tumor. The pathologist described in his report a specimen of total right nephrectomy, which contained residual tumor of medullary carcinoma with extensive fibrous and inflammatory changes related to adjuvant chemotherapy. The therapeutic response was estimated to be 60% (60% fibrosis and 40% viable tumor) of the overall surface area of the macroscopically observed scarred zone, measuring 2.5 cm in its largest dimension. The residual tumor was composed of more-or-less cohesive masses of cells, with abundant eosinophilic cytoplasm that was clarified in places with an irregular nucleus, strongly nucleolated within a myxoid stroma. No sarcomatoid or rhabdoid features were observed. On immunohistochemical examination, these tumor cells expressed cytokeratins 7 and 903, vimentin, and PAX8, but not INI1. PD-L1 was expressed in 5% of tumor cells. The tumor was strictly intrarenal and did not invade the renal vein or small vessels. The collecting system and adrenal gland were not involved. The inter-aortocaval lymph node dissection revealed a metastatic lymph node measuring 4 cm in its largest dimension, without capsular rupture, with fibrous changes, one node measuring 0.3 cm with complete fibrous remodeling in favor of a tumor response, and four negative nodes (one positive out of six nodes). There were microfoci of tumor cells in the periganglionic connective tissue. The surgical margins were clear. Overall, the tumor was classified as ypT1aN1R0 according to the TNM 2018 classification, AJCC/UICC 8th edition. Histologic features are shown in **Figure 2**.

Subsequently, adjuvant therapy under the same cytotoxic chemotherapy protocol was administered for three cycles, and surveillance was conducted until a relapse was detected in October 2020, through 18F-FDG PET/MRI, which revealed progression of the retroperitoneal lymph nodes, with two hypermetabolic lymph nodes (14-mm inter-aortocaval and 10-mm latero-aortic). The case was reviewed at a multidisciplinary meeting, and it was decided that a rechallenge of chemotherapy should be pursued before the possibility of surgical reintervention was contemplated. In the absence of validated therapeutic alternatives, it was decided to administer gemcitabine at 1,250 mg/m^2^ (on days 1 and 8) in association with cisplatin at 70 mg/m^2^ (on day 1), which was reduced to 65 mg/m^2^ after the third cycle due to grade 3 neutropenia. Following three cycles of treatment, a partial response was observed, as evidenced by a reduction in the size of the inter-aortocaval lymph node from 14 mm to 7 mm and the latero-aortic lymph node from 10 mm to 5 mm. Following six cycles, the patient was proposed for surgical management of the remaining retroperitoneal disease, and underwent extensive retroperitoneal lumbo-aortic and aorto-cava lymph node dissection in April 2021. The operative time was 195 min, with an estimated blood loss of 1,300 ml. The histology report indicated malignancy in 11 out of 16 lumbo-aortic nodes (11N+/16), and in 2 out of 10 inter-aorto-cava nodes (2N+/10).

Progression of the disease was diagnosed 10 months after the last surgery: the patient described intense right flank pain that had been present for several weeks, requiring a significant increase in oral morphine doses. The CT scan that was then performed to explore these symptoms showed a reappearance of retroperitoneal lymph nodes without any other identifiable lesions, suggestive of disease progression. The patient’s case was presented at a specialized multidisciplinary consultation meeting to search for molecular anomalies. A liquid biopsy “Foundation One Liquid CDx” was performed but did not allow for inclusion in a therapeutic trial or specific therapeutic orientation [TP53 mutation negative, low tumor mutational burden (TMB at 4 MBs), no loss of heterozygosity, microsatellite stable (MSS)]. The patient was therefore proposed for systemic treatment with carboplatin, doxorubicin, and bortezomib, but he did not wish to resume chemotherapy and requested some time to reflect. Inclusion in the phase I therapeutic trial PEMBIB (17) was proposed, but unfortunately, the patient’s general condition no longer allowed for inclusion in the protocol, with the appearance of symptomatic ascites effusion requiring evacuative punctures.

The patient presented to the emergency department 4 months after being diagnosed with recurrent disease. He reported persistent asthenia for several weeks without significant worsening, but with the gradual appearance of edema in the lower limbs and tense ascites requiring two trans-abdominal punctures for evacuation. A deterioration of the general condition was noted from the end of May 2022, with an inability to eat and the onset of anuria 24 h prior to presentation to the emergency department. He was subsequently admitted to the intensive care unit due to multiorgan failure. A non-enhanced thoracoabdominal-pelvic CT scan was performed due to renal insufficiency: this showed the appearance of multiple hypodense nodular lesions in the liver suggestive of secondary lesions; large retroperitoneal nodules in the right nephrectomy bed; an increase in the number and size of aortocaval, iliac, and inguinal lymph nodes; the appearance of bilateral pleural effusion; and a large amount of intra-abdominal fluid accumulation. The appearance of a lytic lesion of the L2 vertebral body, suggestive of a secondary lesion, was also indicated. Given the severity of the clinical picture and the absence of therapeutic resources, a collective decision (involving oncologists and intensivists) not to perform invasive resuscitation procedures was taken.

**Figure 3:**
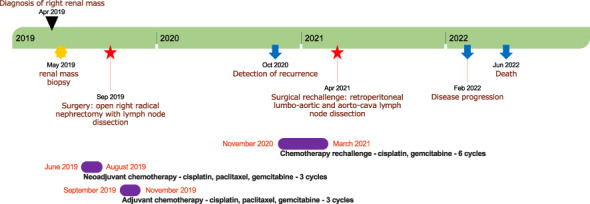
A timeline depicting patient care with the main notable events during follow-up. The timeline is represented by a green stripe divided into years. The black triangle indicates the date of diagnosis; the yellow star denotes the timing of renal mass biopsy; the red stars indicate the timing of surgeries; the blue arrows denote the timing of recurrence and death; and the violet stripes represent periods of chemotherapy.

This patient unfortunately passed away in June 2022, 37 months after initial diagnosis. A timeline depicting patient care is shown in **Figure 3**.

## 3 Discussion

Despite advancements in the understanding of this disease, no significant improvement in disease-specific survival has been observed over the past decade, and treatment is currently based on chemotherapy and nephrectomy, despite a lack of solid scientific evidence (18–20). In 2016, Beckermann et al. proposed clinical guidelines, developed in collaboration with a panel of experts, to aid in the clinical management of these patients (8). These guidelines were subsequently updated in 2019 by Msaouel et al., with a major change in the form of the proposal that nephrectomy should only be performed in the event of a response to chemotherapy in a perioperative scheme (21).

We report a single patient with a prolonged OS of 37 months, in contrast to the data reported by Shah et al., who found a median OS of 16.4 months in 38 patients treated with nephrectomy before or during cytotoxic chemotherapy (6). This highlights the exceptional nature of this patient’s response. The current expert consensus, as reported by Msaouel et al., supports the use of platinum-based cytotoxic chemotherapy as prior systemic therapy for RMC treatment (21). Cisplatin-based chemotherapy (specifically, cisplatin plus gemcitabine in combination with doxorubicin or high-dose MVAC) is the best-described and most effective known treatment for RMC, with an overall response rate (ORR) of 29% according to literature sources (6, 8, 18, 19, 22–24). However, this treatment strategy appears to be based on weak scientific foundations, approaching historical treatments of metastatic upper tract urothelial cancer or collecting duct carcinoma, in the absence of alternative therapies. Patients who demonstrate a radiological response to the disease should be considered for nephrectomy in conjunction with retroperitoneal lymphadenectomy. Systemic therapy should then be continued, with close clinical and radiological follow-up every 6 to 12 weeks.

The supposed benefit of cytoreductive nephrectomy in RMC patients is based on a retrospective study of 52 patients (6). The comparison between patients who underwent nephrectomy and those who received only systemic therapy suggested a benefit in favor of the nephrectomy group, with a median OS of 16.4 months compared to 7.0 months, respectively (*p* < 0.001, HR = 0.22, 95% CI: 0.09–0.51).

With significant advancements in the diagnosis and understanding of this disease having occurred over the past two decades, it is noteworthy that the rarity of RMC may contribute to the lack of scientific evidence regarding its management. Nonetheless, ongoing phase II clinical trials are exploring alternative treatment options based on identified molecular pathways and plausible physiopathological hypotheses. Among these research axes are therapeutics targeting the proteasome, such as bortezomib, which promotes cell death in SMARCB1-deficient tumors (25). Studies evaluating proteasome inhibitors as systemic treatments for RMC have been conducted. For instance, a phase II study published in 2004 assessed bortezomib for patients with advanced RCC and reported clinical activity, with 4 out of 37 evaluable patients showing partial response (11% ORR). Interestingly, only one of the four responders had RMC, despite being the only one with this histology, while the other three had ccRCC (26, 27). In addition, there have been reports of prolonged responses in children with metastatic RMC who were treated with a combination of bortezomib and platinum-based chemotherapy. Two cases of exceptional complete responses with no evidence of disease at 23 months and 7 years from diagnosis were reported by Carden et al. (28). A phase II trial (NCT03587662) is currently recruiting patients to evaluate the safety and efficacy of ixazomib, a second-generation proteasome inhibitor, in combination with gemcitabine and doxorubicin in patients with SMARCB1-negative renal tumors (29).

EZH2 (enhancer of zeste homologue 2) inhibitors are a type of anticancer drug that regulate DNA transcription by inhibiting histone methylation. In mice with SMARCB1-deleted tumors, these inhibitors have been found to be effective in halting histone methylation (30, 31). EZH2 inhibitors work by demethylating lysine 27 of histone H3, which can enhance the effectiveness of other drugs like doxorubicin (32). Through demethylation of histone H3, EZH2 inhibitors can also restore the expression of proapoptotic genes in cancerous cells, promoting programmed death. One particular EZH2 inhibitor, known as tazemetostat, is currently undergoing evaluation in a phase II basket study. The study is recruiting patients with SMARCB1-negative tumors, including refractory synovial sarcoma and RMC patients. Recruitment was active at the time of writing, with an estimated primary completion date of December 2022 (NCT02601950). The authors of the study reported clinical activity in a subset of a cohort of 62 patients treated for epithelioid sarcoma with tazemetostat (800 mg, oral drug, twice daily), with a 15% ORR (95% CI: 7%–26%) (33). Unfortunately, no results on RMC patients treated with tazemetostat in this study have yet been reported.

Lastly, immune-oncology (IO) using anti-PD-1 and anti-CTLA4 antibodies has demonstrated clinical activity in RMC. In one study, Sodji et al. reported clinical activity in a single patient with metastatic RMC who was treated with nivolumab (anti-PD-1 antibody) for 15 months after recurrence following initial nephrectomy and six adjuvant cycles of cisplatin-based chemotherapy (34). The patient experienced stability of retroperitoneal lesions and regression of pulmonary secondary lesions while on nivolumab. The authors did not suggest any predictive role of PD-L1 tumoral expression in response to nivolumab. Three RMC patients were included in a phase I study that evaluated the safety of nivolumab plus cabozantinib (a tyrosine kinase inhibitor) ± ipilimumab (anti-CTLA4 antibody) for patients with metastatic urothelial carcinoma and other genitourinary tumors (35). Two of these patients were evaluated for treatment response, with one achieving a partial response and the other showing progressive disease during treatment. The use of ipilimumab in these patients was not mentioned. Furthermore, another study reported the use of pembrolizumab (an anti-PD-1 antibody) in four RMC patients (36).

Regrettably, it appears evident that the scarcity of RMC patients (who are rare and medically complex) constrains the enrollment of participants in clinical trials and obstructs the successful completion of phase III research. Consequently, it is of paramount importance to incorporate RMC patients in phase II studies, as indicated in the accompanying table (**Table 1**).

**Table 1:** Ongoing phase II trials for RMC.

Trial	Phase	Drugs	Recruitment status	Related publications
Ixazomib, Gemcitabine, and Doxorubicin in Treating Patients With Locally Advanced or Metastatic Kidney Cancer—NCT03587662	II	Ixazomib + doxorubicin + gemcitabine	Recruiting	Msaouel et al. (29)
A Phase II, Multicenter Study of the EZH2 Inhibitor Tazemetostat in Adult Subjects with INI1-Negative Tumors—NCT02601950	II	Tazemetostat	Active, not recruiting	Gounder et al. (33)
Phase II Trial of Nivolumab Plus Ipilimumab in Patients with Renal Medullary Carcinoma—NCT03274258	II	Nivolumab + ipilimumab	Active, not recruiting	Msaouel et al. (37)
Secured Access to Nivolumab for Adult Patients with Selected Rare Cancer Types (AcSe)—NCT03012581	II	Nivolumab	Active, not recruiting	Albiges et al. (38)

## 4 Conclusion

We report here the case of a 31-year-old patient with RMC who received appropriate treatment consisting of initial cisplatin-based chemotherapy, followed by nephrectomy with retroperitoneal lymphadenectomy and continued adjuvant chemotherapy. He survived for 37 months after the initial diagnosis, supporting the therapeutic strategy employed here based on expert consensus. However, novel therapies are urgently needed to improve outcomes for patients with this rare and life-threatening disease. Ongoing phase II clinical trials are currently recruiting RMC patients.

## Data availability statement

The original contributions presented in the study are included in the article/supplementary material. Further inquiries can be directed to the corresponding author.

## Ethics statement

Written informed consent was obtained from the individual(s) for the publication of any potentially identifiable images or data included in this article.

## Author contributions

Study concept and design: JC, AT, AI. Drafting of the manuscript: JC, AI, RC, MMam, RB, DA, SE, OK, MMar, NP, and ER. Critical revision of the manuscript: AT, AI, and MMam. All authors contributed to the article and approved the submitted version.
